# Transmembrane proteins with unknown function (TMEMs) as ion channels: electrophysiological properties, structure, and pathophysiological roles

**DOI:** 10.1038/s12276-024-01206-1

**Published:** 2024-04-01

**Authors:** Hyunji Kang, C. Justin Lee

**Affiliations:** Center for Cognition and Sociality, Life Science Cluster, Institute for Basic Science (IBS), 55 Expo-ro, Yuseong-gu, Daejeon, 34126 Republic of Korea

**Keywords:** Physiology, Diseases

## Abstract

A transmembrane (TMEM) protein with an unknown function is a type of membrane-spanning protein expressed in the plasma membrane or the membranes of intracellular organelles. Recently, several TMEM proteins have been identified as functional ion channels. The structures and functions of these proteins have been extensively studied over the last two decades, starting with TMEM16A (ANO1). In this review, we provide a summary of the electrophysiological properties of known TMEM proteins that function as ion channels, such as TMEM175 (K_EL_), TMEM206 (PAC), TMEM38 (TRIC), TMEM87A (GolpHCat), TMEM120A (TACAN), TMEM63 (OSCA), TMEM150C (Tentonin3), and TMEM43 (Gapjinc). Additionally, we examine the unique structural features of these channels compared to those of other well-known ion channels. Furthermore, we discuss the diverse physiological roles of these proteins in lysosomal/endosomal/Golgi pH regulation, intracellular Ca^2+^ regulation, spatial memory, cell migration, adipocyte differentiation, and mechanical pain, as well as their pathophysiological roles in Parkinson’s disease, cancer, osteogenesis imperfecta, infantile hypomyelination, cardiomyopathy, and auditory neuropathy spectrum disorder. This review highlights the potential for the discovery of novel ion channels within the TMEM protein family and the development of new therapeutic targets for related channelopathies.

## TMEM channels: potential therapeutic targets for various disorders

Transmembrane proteins (proteins that stretch across the cell membrane) play a role in many cell functions. Yet, we don’t fully understand the structure and roles of many of these proteins. Recent research has found that some transmembrane proteins, like TMEM16A, TMEM87A, TMEM150C, TMEM63, TMEM43, and TMEM175, act as ion channels (pathways for charged particles) crucial for various bodily functions. These proteins participate in normal and abnormal bodily processes, and when they don’t work properly, they can cause diseases like Parkinson’s disease, cancer, brittle bone disease, and hearing loss. By understanding these proteins better, we could develop new treatments for these diseases. Future studies should aim to understand exactly how these proteins work and their potential as targets for treatment. This summary was initially drafted using artificial intelligence, then revised and fact-checked by the author.

## Introduction

The transmembrane (TMEM) protein family is a group of membrane-spanning proteins localized to the plasma membrane or intracellular organelle membranes; these proteins include those of mitochondria, lysosomes, endosomes, the endoplasmic reticulum, and Golgi membranes^1^. TMEM proteins are numbered and not named since the transmembrane domains in their protein sequence have been identified; however, their structure and biological functions have not been determined. After extensive characterization of their functions, many TMEM proteins were recently renamed and reclassified into specific categories, such as ion channels, transporters, or G protein-coupled receptors^2^.

**Fig. 1:**
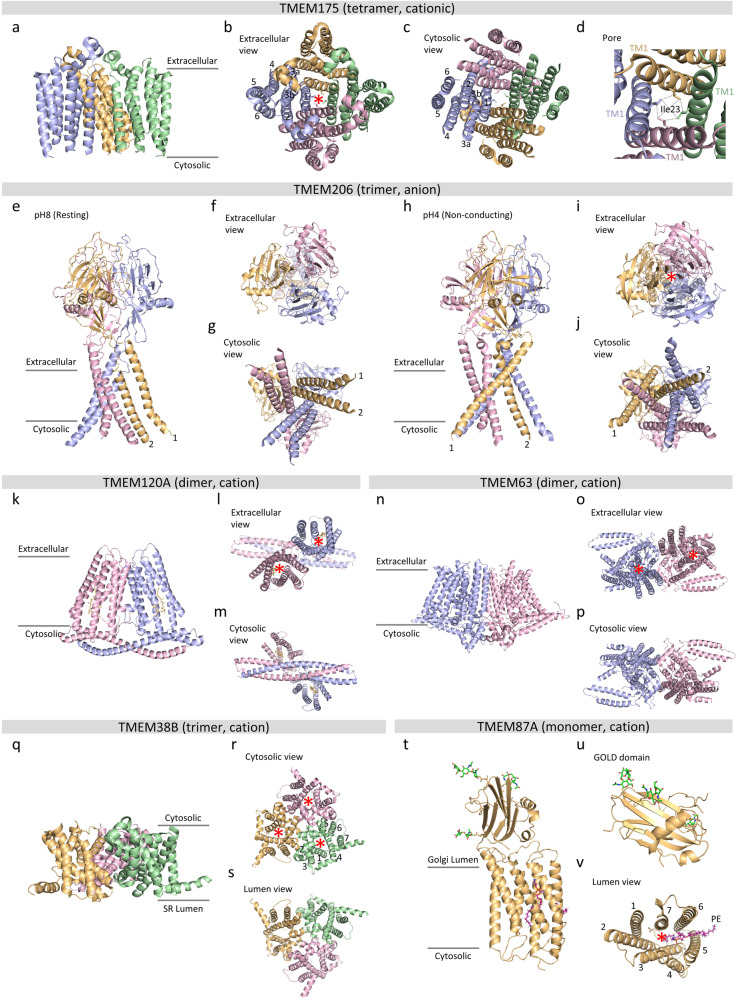
Representative structures of each TMEM channel. Representative structures of each TMEM channel. Red asterisks indicate channel pore(s). **a** Side view of TMEM175 (K_EL_) (PDB; 5VRE) with subunits. Tetrameric structure of TMEM175 (K_EL_) from the extracellular (**b**) and cytosolic (**c**) images. **d** Zoomed-in view of the pores of TMEM175 (K_EL_). Side view of TMEM206 (PAC) (PDB; 7JNA and 7JNC) at pH 8 (**e**) and pH 4 (**h**). Trimeric structure of TMEM206 (PAC) from the extracellular (**f**, **i**) and cytosolic (**g**, **j**) perspectives at pH 8 and pH 4, respectively. **k** Side view of TMEM120A (TACAN) (PDB; 7F3T) with the CoASH complex. Dimeric structure of TMEM120A (TACAN) from the extracellular (**l**) and cytosolic (**m**) regions. **n** Side view of TMEM63 (OSCA) (PDB; 6JPF). Dimeric structure of TMEM63 (OSCA) from the extracellular (**o**) and cytosolic (**p**) views. **q** Side view of TMEM38B (TRIC-B) (PDB; 6JYX). Trimeric structure of TMEM38B (TRIC-B) from the extracellular (**r**) and cytosolic (**s**) views. **t** Side view of TMEM87A (GolpHCat) (PDB; 8HSI). **u** Extracellular GOLD domain of TMEM87A (GolpHCat). **v** Lumen view of TMEM87A (GolpHCat) with PE.

Among the 272 TMEM proteins^3^, 9 have been extensively studied, classified, and renamed as functional ion channels, which facilitate ion transport across the membrane, including TMEM16A^4–6^ (ANO1), TMEM175^7^ (K_EL_), TMEM38^8^ (TRIC), TMEM206^9^ (PAC), TMEM87A^10,11^ (GolpHCat), TMEM120A^12^ (TACAN), TMEM63^13^ (OSCA), TMEM150C^14^ (Tentonin3), and TMEM43^15^ (Gapjinc), along with some crystallography or cryogenic electron microscopy (Cryo-EM) structures, including TMEM16A^16,17^ (ANO1), TMEM175^18^ (K_EL_), TMEM38^19^ (TRIC), TMEM206^20^ (PAC), TMEM87A^21^ (GolpHCat), TMEM120A^22^ (TACAN), TMEM63^23^ (OSCA) (Fig. 1 and Table 1). In this review, we describe the electrophysiological properties and structures of these TMEM proteins, excluding TMEM16A because of the abundance of review papers covering the structure of the channel present in literature^16,24^ and the large number of pathologies associated with the channel^25–29^, which cannot be covered by this review alone. The purpose of this review is to provide inspiration for new discoveries of ion channels with unique architectures among the remaining TMEM proteins.

**Table 1:** Summary of TMEM proteins as ion channels: name, cellular localization and structure information.

Name	Channel	Localization	Structure (RCSB Protein Data Bank)	Reference
TMEM175 (K_EL_)	Cation channel	Lysosomal membrane	SVRE, 6HD8, 6HD9, 6HDA, 6HDB, 6HDC, 6SWR, 6WC9, 6WCA, 6WCB, 6WCC,	^18,31,32^
TMEM206 (PAC)	Proton-activated chloride channel	Plasma membrane	7JNA, 7JNC, 7JI3, 7SQG, 7SQH, 7SQF, 8EQ4, 8FBL	^20,47,48^
TMEM38 (TRIC)	Monovalent selective cation channel	Sarcoplasmic reticulum (SR) or endoplasmic reticulum (ER) membrane	5EGI, 5EIK, 5WUC, 5WUD, 5WUE, 5WUF, 5WTR, 6IYU, 6IYX, 6IZF, 6IYZ, 6IZ0, 6IZ1, 6IZ5, 6IZ3, 6IZ4, 6IZ6	^19,54,56–58^
TMEM87A (GolpHCat)	Non-selective cation channel	Golgi membrane	8CTJ, 8HTT, 8HSI	^21,73^
TMEM120 (TACAN)	Mechanosensitive ion channel	Nuclear envelope membrane, plasma membrane	7F3U, 7F3T, 7N7P, 7N0K, 7N0L, 7CXR, 7F73, 7F6V	^12,76,77^
TMEM63 (OSCA)	Hyperosmolarity-gated calcium-permeable channel	Plasma membrane	5Z1F, 6JPF, 6MGV, 6MGW	^23,86–88^
TMEM150C (Tentonin3)	Mechanosensitive ion channel	Plasma membrane	None	^14^
TMEM43 (Gapjinc)	Non-selective cation channel	Nuclear envelope, ER, plasma membrane	None	^15^

**Fig. 2:**
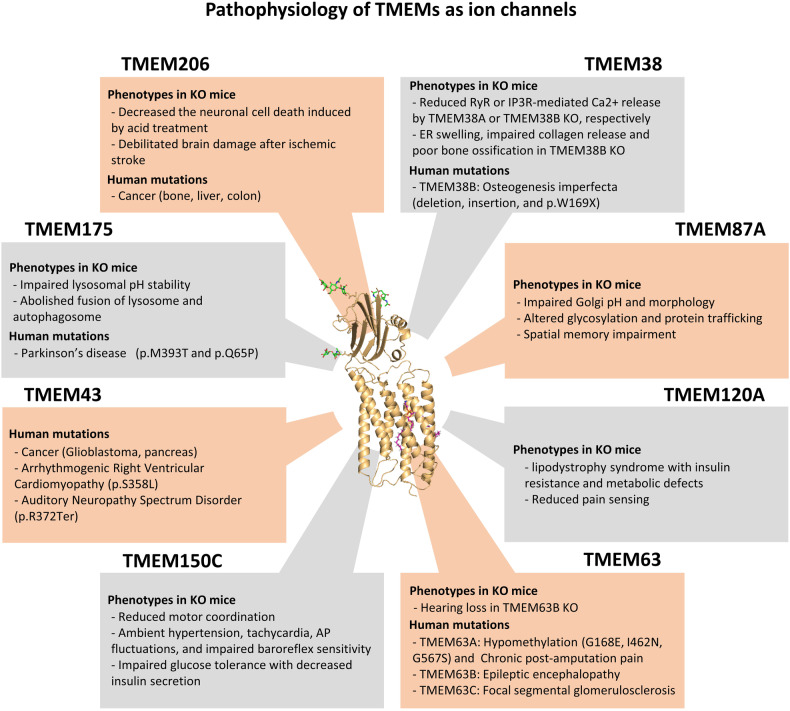
Summary of the physiological and pathophysiological properties of TMEM proteins as ion channels.

Ion channels play crucial roles in various physiological processes, including maintenance of membrane potential homeostasis, regulation of intracellular pH, and facilitation of transmitter release. Dysfunction in ion channel subunits, whether due to gain or loss of function^30^, leads to abnormal physiological processes and channelopathies. In this review, we describe the physiological and pathophysiological functions of TMEM proteins (Fig. 2) and provide deep insights into newly discovered TMEM protein-related channelopathies. By doing so, we aim to suggest potential therapeutic molecular targets for individuals afflicted by TMEM protein-related channelopathies.

### TMEM175 (K_EL_)

#### Electrophysiological properties

TMEM175 has been recognized as a lysosomal membrane protein with an unknown function through proteomic analysis via tandem mass spectrometry (LC–MS/MS)^31,32^. TMEM175 has no sequence homology with any other functionally known membrane proteins and was renamed TMEM175 after being identified as a constitutively active leak-like potassium channel, as confirmed by direct recordings of lysosomal K^+^ conductance^7^. In addition, it mediates a proton current. It has also been reported that the K^+^ current through TMEM175 decreases, while the proton current increases with decreasing pH^33^.

Prokaryotic TMEM175 contains a single domain with six transmembrane helices (6TMs), whereas mammalian TMEM175 has two 6TM domains^7^. TMEM175 in both prokaryotic and mammalian species has phylogenetically no sequence homology with canonical tetrameric K^+^ channels and lacks the TVGYG selectivity filter motif observed in K^+^ channels such as KcsA and Shaker channels^7,34,35^. Canonical K^+^ channels exhibit higher selectivity for K^+^ than for Cs^+^ and immeasurably lower permeability to Na^+^^34^. In contrast, mammalian TMEM175 exhibits the highest permeability for Cs^+^, followed by K^+^ and Na^+^, whereas prokaryotic TMEM175 shows low selectivity for K^+^ and similar permeability to both Cs^+^ and K^+^^7,18,36^. The physiological properties of TMEM175 thus, reflect its uniqueness and allow us to predict that the structure, ion permeation and selectivity mechanism of this channel differ from those of canonical K^+^ channels.

#### Structure

The structure of the prokaryotic TMEM175 channel has been revealed for *Chamaesiphon minutus* (CmTMEM175)^18^ and *Marivirga tractuosa* (MtTMEM175)^36^; the structure of the eukaryotic TMEM175 channel has been revealed for *Homo sapiens* (HsTMEM175)^37^. Prokaryotic TMEM175 has a homotetrameric architecture, with each subunit harboring 6TM domains (Fig. 1a–c)^18^. In contrast, eukaryotic TMEM175 has a homodimeric architecture with each subunit harboring two 6TM domains^37^. In prokaryotic TMEM175, four TM1 helices form a pore-lining inner helix that can create an ion permeation pathway^18^. Similarly, four TM domains in eukaryotic TMEM175, two TM1 and two TM7 domains, form an ion conduction pore^37^. The pore contains the hydrophobic residue Ile23 in each TM1 helix, forming a bottleneck in prokaryotic TMEM175^18,36^ (Fig. 1d). Ile23 in Tm1 of CmTMEM175 and Ile46 and Ile271 in TM1 and TM7 of HsTMEM175 form the gating mechanism for dehydrated K^+^ through these channels^18,37^. Additionally, both the N-terminus of the cytosolic entrance and the C-terminus of the extracellular entrance enclose the negatively charged surface potential^18^, which may be necessary for cation accumulation at the entrance of the pore lining. Furthermore, the RxxxFSD signature sequence motif in TM1 and TM7 plays a crucial role in tetrameric and dimeric assembly via inter- and intrasubunit interactions, respectively, for TMEM175 channel functions^18,36,37^. Hence, the overall structures of prokaryotic and eukaryotic TMEM175 proteins, including those of the ion conduction pore and ion conduction pathway, are similar.

#### Physiological and pathophysiological roles

The lysosomal lumen is maintained at pH 4.5–5 and mediates crucial cellular processes, including autophagy, endocytosis, and phagocytosis. The lysosomal membrane potential should be regulated by numerous channels and transporters embedded in the membrane to maintain acidic conditions, and defects in these proteins lead to various diseases^38–41^. TMEM175 has been identified as a lysosomal leak-like K^+^ channel with the function of determining lysosomal membrane potential at rest and regulating lysosomal pH stability and fusion of lysosomes and autophagosomes during autophagy^7^. These functions are dysregulated in the lysosomes of TMEM175 KO mice^7^, indicating that TMEM175 is critical for maintaining lysosomal homeostasis.

Genome-wide association studies (GWASs) suggest that TMEM175 is associated with Parkinson’s disease^42–44^. TMEM175 deficiency results in an unstable lysosomal pH, which leads to decreased lysosomal catalytic activity and impaired autophagosome clearance^45^. Furthermore, impaired autophagy due to knockdown of TMEM175 leads to increased aggregation of phosphorylated α-synuclein, which is a key reason for the α-synucleinopathy observed in Parkinson’s disease in primary rat hippocampal neurons^45^. Recently, the missense single nucleotide polymorphism (SNP) rs34311866 (p.Met393Thr) in TMEM175 has been studied as a risk factor for Parkinson’s disease^46–49^. Structurally, Met393Thr is in TM4, and the side chain of methionine faces toward the inside of the TM domain^48^. The risk variant Met393Thr is predicted to reduce the stability of the hydrophobic core of the TM domain and cause folding impairment, resulting in decreased activity of TMEM175 as an ion channel without affecting its localization to the lysosomal membrane^46^. Functionally, TMEM175 Met393Thr reduces the stability of the lysosomal pH and autophagosome clearance and increases phosphorylated α-synuclein aggregation, similar to the phenotype of TMEM175 KO^47^. Recently, Wie et al. discovered that TMEM175 forms a complex with protein kinase B (AKT), which is activated by growth factor and called lysoK_GF_^48^, and this complex is required for the functional activity of the TMEM175 channel. TMEM175 Met393Thr causes lysoK_GF_ loss of function and reduces channel activity. Moreover, this variant induces accumulation of α-synuclein and accelerates cognitive and motor decline in Parkinson’s disease^48^. In contrast, another common variant, Gln65Pro, of TMEM175 (rs3488217) increases channel activity, protects neurons and decreases the risk of developing Parkinson’s disease^48^. In summary, recent findings reveal the role of TMEM175 as a lysosomal membrane ion channel and that loss-of-function TMEM175 variants, which are associated with Parkinson’s disease, may be attractive therapeutic targets for treating this disease. Future studies should be conducted to determine whether Parkinson’s disease symptoms appear in TMEM175 KO or point-mutant mice.

### TMEM206 (PAC)

#### Electrophysiological properties

Two independent research teams have identified TMEM206 through genome-wide RNA interference (RNAi) screens, identifying it as a crucial acid-sensitive outwardly rectifying anion channel (PAORAC/ASOR) and subsequently renaming it as PAC due to its role as a proton-activated chloride channel^9,50^. TMEM206 is present not only in the plasma membrane but also in the endosomal membrane^9,50,51^. It has two TM domains, and both the N- and C-termini face the cytosolic sides^9^. It has been demonstrated that endogenous TMEM206 is an essential component of proton-activated Cl^-^ currents (*I*_*Cl,H*_) via CRISPR‒Cas9 genomic editing in HEK293 cells^9,50^. The order of permeability of human TMEM206 (hPAC/hTMEM206) is SCN^−^ > I^−^ > NO_3_^−^>Br^−^>Cl^−^, which is the same as that of the native *I*_*Cl,H*_. The hTMEM206-mediated current is efficiently blocked by 4,4′diisothiocyano-2,2′-stilbenedisulfonic acid (DIDS), niflumic acid (NFA), and 5-nitro-2-(3-phenylpropylamino)benzoic acid (NPPB), which are nonspecific *I*_*Cl,H*_ inhibitors^9,50^. The TMEM206 protein in all vertebrates is sensitive to pH and mediates *I*_*Cl,H*_ but has different biophysical properties, such as permeability order and ion selectivity^9,50^. Taken together, these findings indicate that TMEM206 has properties and features similar to those of endogenous proton-activated chloride channels.

#### Structure

Recently, the cryo-EM structure of hTMEM206 was studied by two different research groups^20,52^. As an ion channel, it has homotrimeric features, with each subunit containing an extracellular domain (ECD) and two transmembrane helices (TM1 and TM2; Fig. 1e–j)^20,52^. Ruan et al. revealed the structures of hTMEM206 at high pH (pH 8.0) and low pH (pH 4.0), indicating a closed state and a proton-bound nonconducting state, respectively^20^ (Fig. 1e–j). However, Wang et al. reported an activated open-state structure at pH 4.5 and suggested that the previously reported pH 4.0 structure represents a desensitized state of the TMEM206 channel^52^. In summary, at the structural level, hTMEM206 is closed at pH 8.0, open at pH 4.5, and desensitized at pH 4.0 in a proton-bound nonconducting state.

The conformational change in the extracellular domain-transmembrane domain interface results in the formation of an acidic pocket under acidic conditions^20,52^. His98 was proposed by Ruan et al. as a key residue for the pH-sensing mechanism due to its presence in this acidic pocket at pH 4.0^20^. In contrast, Wang et al. concluded that His98 is not important in the activated state and that six negatively charged residues function as pH sensors when protonated in the acidic pocket^52^. It is possible that the discrepancy between the two groups (Ruan et al. and Wang et al.) is because the structures were resolved at pH 4.0 and pH 4.5, respectively.

Hydrophobic residues, such as Ala318 and Leu315 on TM2, form the narrowest constriction in the closed state, whereas the ion-conduction pore is formed by Gly312, Leu315, and Ala316 on the TM2 subunits in an activated state^52^. The positively charged Lys319 residue within the ion-conducting pore determines the anion selectivity of hTMEM206. When replaced with negatively charged glutamic acid (Lys319Glu), the anion selectivity converts to cation selectivity for the hTMEM206 channel with an inwardly rectifying current^20^. The structure of pufferfish TMEM206 is similar to that of hTMEM206. It also forms a trimeric channel, and the pore is lined by hydrophobic residues, such as Gly313 and Met316, on TM2. Moreover, Lys320, corresponding to Lys319 in hTMEM206, is the determinant of anion selectivity in pufferfish TMEM206^53^.

Interestingly, the topology, structure, and assembly of TMEM206 are similar to those of acid-sensing ion channels (ASICs) and epithelial sodium channels (ENaCs), despite undetectable amino acid sequence homology^53^. This example raises the interesting possibility that many unknown ion channels might present structures similar to those of already known channels.

#### Physiological and pathophysiological roles

TMEM206 traffics from the plasma membrane to the endosomal membrane. Endosomal TMEM206 channels sense low pH and prevent hyperacidification by releasing Cl^-^ from the lumen^51^. Local acidosis mediated by acid-sensing ion channels is a hallmark of many diseases, such as ischemia and cancer. Wang et al. previously proposed that the ASOR anion channel, which is sensitive to DIDS and phloretin, is involved in acidosis-induced cell death^54^. However, the molecular identity of ASOR remains undetermined^54^. Recently, it has been suggested that TMEM206 is an ASOR channel that essentially plays a role in acid-induced cell death in HEK293 cells. In support of this idea, TMEM206 KO cells are partially protected from cell death triggered by acid treatment^9,50^. Genetic deletion of TMEM206 in primary rat cortical neurons abolishes proton-activated Cl^-^ currents and decreases the neuronal cell death induced by acid treatment. Additionally, TMEM206 KO causes debilitating brain damage in mice after ischemic stroke^9,55^. However, studies of the association of TMEM206 with ischemia in humans are lacking.

Furthermore, TMEM206 has been studied in many types of cancer, including bone, liver, and colon cancer^56–58^. In colorectal cancer, expression of the TMEM206 mRNA and protein is upregulated, and TMEM206 interacts with AKT and alters ERK levels, promoting proliferation and migration^56^. Increased TMEM206 expression is associated with poor prognosis in patients with hepatocellular carcinoma^57^. It has been demonstrated that TMEM206 is upregulated and, together with its downstream target β-catenin, modulates tumor growth and metastasis in osteosarcoma^58^. Although TMEM206 is associated with various cancers, no studies have been conducted to determine how TMEM206, a proton-activated Cl^-^ channel, affects cancer progression. Once the precise underlying mechanisms are determined, TMEM206 can be studied as a potential therapeutic target in cancer treatment.

### TMEM38 (TRIC)

#### Electrophysiological properties

TMEM38 is an intracellular monovalent selective cation channel named after a trimeric intracellular cation-selective channel (TRIC). Vertebrate TMEM38s exist in two distinct paralogous TMEM38s, namely, TMEM38A and TMEM38B. The orthologous TMEM38A proteins have ~50 to 70% pairwise sequence similarity, whereas the orthologous TMEM38B proteins are slightly more divergent, sharing ~40% sequence identity^59^. Mammalian TMEM38A is expressed in excitable tissues such as the muscle and brain and localizes to the sarcoplasmic reticulum membrane, whereas TMEM38B is expressed in the endoplasmic reticulum of most mammalian tissues at relatively low levels^8^. Reconstitution of purified TMEM38A and TMEM38B from skeletal muscle results in single-channel conductance with higher selectivity for K^+^ than for Na^+^ but not for divalent cations^8,60^. Different activation mechanisms of both channels have been suggested^60^. TMEM38A is opened by a change in voltage, such as negative potential, within the sarcoplasmic reticulum lumen. TMEM38B is activated by increased cytosolic Ca^2+^ levels and by changes in voltage, and this process is inhibited when luminal Ca^2+^ levels increase^60^. Unlike vertebrate TMEM38 proteins, invertebrate and prokaryotic TMEM38 proteins do not exhibit such diversity in terms of intracellular distribution, activating mechanisms, or functions.

#### Structure

High-resolution structures of TMEM38 channels from prokaryotes^61–63^, invertebrates^19^, and vertebrates^59^ have been reported. All TMEM38s, regardless of species or subtype, form a symmetrical homotrimeric complex (Fig. 1q–s). Each protomer contains an hourglass-shaped pore composed of seven TM helices^19,59,63^ and has a novel structure, such that the 2 domains, containing 3 helices each, TM1-3 and TM4-6, lie antiparallel to each other, despite low sequence homology^61,62^. Lateral fenestrations allowing for entry of lipid molecules into the protein structure have been found at protomer interfaces in prokaryotic, invertebrate and vertebrate TMEM38 proteins^59,61^. Both prokaryotic and eukaryotic TMEM38s have highly conserved glycine residues within TM2 and TM5, generating kinks in the middle of the pore region. These kinks lead to channel opening and may affect channel activity^19,59,61–63^. Prokaryotic and eukaryotic TMEM38s have similar overall structures but differ in details^19,59,61–63^. First, lipid molecules, such as PIP2 and DAG, which are known to regulate channel activity, bind to the interface between protomers in eukaryotic TMEM38 channels, whereas neutral lipids bind to these channels in prokaryotes. Second, the calcium-binding site in the lumen is present only in eukaryotic TMEM38. Finally, the voltage dependency of channel opening in eukaryotic TMEM38 relies on the conserved lysine and arginine residues in the TM4 domain, whereas these residues are not conserved in prokaryotic TMEM38^19,59,61–63^. Therefore, eukaryotic and prokaryotic TMEM38 may have different functions because of these structural differences.

#### Physiological and pathophysiological roles

Calcium release by the ryanodine receptor and IP3 receptor from the intracellular sarcoplasmic reticulum/endoplasmic reticulum is required for cellular signaling. Countermovement of ions is also essential for balancing the transient negative membrane potential due to calcium release^64–66^, but the molecular identity of the counterion channel remains unknown. TMEM38 has been reported to play a physiological role in intracellular stores as a counterion channel for calcium handling^8^. Intriguingly, TMEM38A channels may selectively support ryanodine receptor-mediated calcium release in muscle cell types, but TMEM38B channels may maintain IP3 receptor-mediated calcium release in nonexcitable cell types^67–69^. TMEM38B-deficient mice exhibit various phenotypes, including reduced Ca^2+^ release from the endoplasmic reticulum, associated with endoplasmic reticulum swelling, impaired collagen release, and poor bone ossification^70^.

A series of genetic variations in TMEM38B have been found in autosomal recessive osteogenesis imperfecta, which is a rare disorder characterized by increased bone fragility, decreased bone mass, and recurrent bone fractures^71–74^. This variant was first reported in Bedouin families in Israel and Saudi Arabia; it contains a deletion of exon 4 and a surrounding intronic sequence^71^. A second deletion mutation, involving exons 1 and 2 of TMEM38B, was identified in a female Albanian child^72^. Homozygosity for two point mutations in TMEM38B, including a novel acceptor splice site variant in intron 3 leading to the insertion of two amino acids (p.Arg151_Gly152insValLeu) and a nonsense mutation in exon 4 (p.Trp169X), was recently reported in three probands from nonconsanguineous Han Chinese families^73^. Although bone-related disorder phenotypes have been observed in TMEM38B KO mice, further studies using mice harboring these three genetic mutations are needed to validate the therapeutic efficacy of these mutations in human patients of osteogenesis imperfecta.

### TMEM87A (GolpHCat)

#### Electrophysiological properties

TMEM87A has been reported to be involved in protein trafficking localized mainly to the Golgi apparatus^75,76^, whereas a fraction of human TMEM87A localizes to the plasma membrane in cancer cells such as melanoma^10^ and glioblastoma multiforme^77^. Indeed, TMEM87A, also known as Elkin1, has been proposed to be an ion channel required for mechanically activated currents and an essential component of a novel PIEZO-independent mechanoelectrical transduction pathway^10^. However, this proposal has been immediately contradicted by the lack of mechanosensitive channel activity in proteoliposomes reconstituted with TMEM87A^21^. Moreover, it has not been clearly determined whether this protein is pore-forming or an auxiliary subunit for a mechanosensitive ion channel^10^. Recently, TMEM87A, renamed GolpHCat, was reported to mediate voltage- and pH dependent, inwardly rectifying currents in heterologous systems^11^. TMEM87A is a nonselective cation channel, and its currents are potently blocked by gluconate and the nonselective cation channel blocker gadolinium^11^. Furthermore, single-channel activities with unique voltage-dependent current and ion permeation properties have been demonstrated for proteoliposomes reconstituted with TMEM87A^11^. Thus, TMEM87A is a bona fide ion channel that can be activated by voltage and modulated by pH but not by mechanical stimulation.

#### Structure

Two independent groups have determined the Cryo-EM structure of human TMEM87A (hTMEM87A) in lipid nanodiscs or in the presence of lauryl maltose neopentyl glycol and cholesterol^21,78^. Both groups reported that TMEM87A displays a monomeric, not tetrameric, architecture, which is in contrast to what was previously predicted because it contains a GYG sequence, which is a signature selectivity filter of classical K^+^ channels^21,78^. Both groups agreed that TMEM87A contains a Golgi dynamics (GOLD) domain and membrane-spanning seven transmembrane (7TM) helix domains^21,78^ (Fig. 1t, u). However, the two groups drew profoundly contradictory conclusions regarding the structural determination of TMEM87A as an ion channel. Hoel et al. concluded that TMEM87A may not function as a mechanosensitive ion channel but is structurally related to GOLD domain seven-transmembrane helix (GOST) proteins, which are known to play a role in membrane protein trafficking^21^. In contrast, Han et al. proposed that TMEM87A may function as an ion channel that has a putative ion conduction pathway that consists of negatively charged luminal vestibule (NLV) and constriction sites^78^. Although the structure of hTMEM87A differs from that of TRIC channels, three conserved basic residues (Lys304, Arg305, and Arg309) lining the hTMEM87A TM3 engage with an electronegative phosphatidylethanolamine (PE) head group (Fig. 1v), which closely resembles the voltage-sensing TM4 helix of TRIC-B1^78^. These findings match the proteoliposome patch data, which revealed single-channel activities with unique voltage-dependent currents. Thus, TMEM87A is most likely a pore-forming ion channel that better qualifies as the proposed name GolpHCat, a Golgi-resident, pH-dependent cationic channel, than Elkin1.

#### Physiological and pathophysiological roles

TMEM87A was initially reported to regulate cell migration and cell-to-cell interaction strength through a PIEZO1-independent mechanoelectrical transduction pathway in melanoma cells^10^. According to the Brain RNA-seq database, TMEM87A is highly expressed in the brain cells, such as neurons and astrocytes^79,80^. TMEM87A contributes to Golgi pH homeostasis as a cation channel localized to the Golgi apparatus in human astrocytes^11^. TMEM87A knockout mice exhibit fragmented Golgi morphology and altered glycosylation and protein trafficking in the hippocampus, leading to impaired long-term potentiation and spatial memory^11^. Furthermore, both astrocytic and neuronal TMEM87A are critical for hippocampal spatial and contextual memory^11^. These findings raise the possibility that TMEM87A may be a key molecular protein associated with fragmented Golgi phenotypes observed in neurodegenerative diseases. Future experiments are needed to assess this exciting possibility.

### TMEM120A (TACAN)

#### Electrophysiological properties

Although TMEM120A was initially proposed to be a mechanically sensitive ion channel, conflicting results were subsequently reported. Beaulieu-Laroche et al. first suggested that TMEM120A is an ion channel activated by mechanical forces and renamed this channel TACAN, which means “movement” in Farsi, the Persian language^12^. TMEM120A is highly expressed in dorsal root ganglia (DRGs) and nociceptors^12^. Heterologous expression of TMEM120A increases mechanically evoked currents in cell lines and even in the Piezo1 KO cell line^12^. Upon reconstitution, purified TMEM120A forms an ion-conducting pore to mediate single-channel currents, which are abolished by known blockers of mechanosensitive channel currents, gadolinium and GsMTx4. The authors subsequently reported that TMEM120A contributes to the ultra-slowly adapting MS currents in nociceptive neurons^12^. In contrast, *Parpaite* et al., *Niu* et al. and Del Rosario. et al. suggested that TMEM120A is not an ion channel^81,82^. Parpaite et al. showed that TMEM120A does not mediate slowly/ultra-slowly adapting (SA/ultra-SA) MS currents by showing that it is not expressed in SA/ultra-SA MS current-expressing neurons via single-cell transcriptomic analysis^81^. Niu et al. showed that reconstituted TMEM120A at high protein concentrations produced heterogeneous conduction but did not produce mechanically evoked currents when expressed in HEK cells. Similarly, Del Rosario. et al. reported that TMEM120A does not affect the portion of SA mechanically activating (MA) currents but increases rapidly adapting (RA) MA currents by suppressing Piezo2 channel activity^82^. Hence, one group suggested that TMEM120A may be a mechanically activated ion channel by itself, while two other groups concluded that TMEM120A may not be a mechanically activated ion channel mediating ultra-SA MS. To resolve the conflict among these groups, additional experiments need to be performed to strengthen these claims. By showing the absence of any other pore-forming protein in the purified solutions used by *Beaulieu-Laroche* et al., using liquid chromatography‒mass spectrometry would greatly strengthen the claim based on their data from the reconstitution of purified TMEM120A. In contrast, if *Niu* et al. could show no mechanosensitive currents mediated by TMEM120A in not only soy L-α-phosphatidylcholine (soy-PC) but also different lipid combinations, such as phosphoethanolamine (PE), phosphoglycerol (PG), or phospho-L-serine (PS), for proteoliposome reconstitution, their claims would be strengthened. Future experiments are needed to resolve these conflicts.

#### Structure

Three independent structural biology research teams have reported that they are not convinced whether TMEM120A is a mechanosensitive ion channel because no channel activity by mechanical force was observed in heterologous systems or reconstituted proteoliposomes^22,83,84^. TMEM120A forms a symmetric homodimer (Fig. 1k–m) that has no structural evidence of being an ion channel. Unexpectedly, the 6TM domain in each protomer forms an α-barrel tunnel with a pocket that serves as a binding site for the coenzyme A molecule (CoASH), as confirmed by mass spectrometry. Furthermore, the TM domain of TMEM120A has structural homology to that of the fatty acid elongase ELOVL7, despite low sequence homology between them^22,83,84^. Four histidine residues in the tunnel of ELOVL7 are important for its catalysis and interaction with eicosanoyl-CoA^83^. Two histidine residues, His196 and His197, are conserved in TMEM120A and influence its binding with CoASH^83^. Trp193 of TMEM120A, which is located at the same location as one of the four histidine residues in ELOVL7, affects the binding affinity of CoASH^84^. Therefore, the three groups concluded that the structure of TMEM120A is not consistent with that of an ion channel but rather has a different role as an enzyme for fatty acid metabolism. However, it has been recently proposed that the previously reported structure of TMEM120A is of its closed state due to blockage of the ion conduction pathway caused by CoASH binding to the tunnel^85^. It has been suggested that Met207 in TM3 and Phe223 in TM4 may be key residues for ion conduction by TMEM120A^85^. Taken together, similar to the electrophysiological data, the structural data suggest that TMEM120A is an ion channel. Structural determination of CoASH-binding site-mutant forms may be helpful for mimicking the open state of TMEM120A as an ion channel and investigating the gating mechanism. To validate that TMEM120A is truly an enzyme rather than an ion channel, additional comprehensive functional studies of its fatty acid metabolism-related activity should be conducted in the future.

#### Physiological and pathophysiological roles

Previously, TMEM120A was identified as a nuclear envelope protein 29 by proteomic screening^86^. It is expressed in adipose tissue and plays a crucial role in adipocyte differentiation and lipid metabolism^87^. Adipocyte-specific TMEM120A conditional KO mice exhibit disruption of genome organization within fat stores, resulting in lipodystrophy syndrome with insulin resistance and metabolic defects^88^. TMEM120A is also expressed in sensory neurons, DRG and nonpeptidergic nociceptors^12^. Nociceptor-specific inducible conditional KO of TMEM120A reduces nociceptor mechanosensitivity and behavioral responses to painful mechanical stimuli^12^. This phenotype is attributed to the contribution of TMEM120A to mechanosensitive currents in nociceptors and mechanical pain sensing^12^. With all these in mind, it remains to be examined if these defects in lipid metabolism and mechanical pain sensitivity observed in TMEM120A conditional KO animals are due to its role as an ion channel or an enzyme.

### TMEM63 (OSCA)

#### Electrophysiological properties

OSCA1 has been proposed to be an osmosensor and a newly identified hyperosmolality-gated calcium-permeable channel in plants^13^. OSCA1.1 and OSCA1.2 from *Arabidopsis thaliana* are activated by mechanical stimulation in heterologously expressed PIEZO1-KO HEK cells^89^. OSCAs mediate noninactivating high-threshold nonselective cationic currents with a slight permeability to chloride ions^89^. These OSCA-mediated stretch-activated currents are blocked by gadolinium^89^. Furthermore, reconstituted OSCA1.2 in liposomes displays stretch-activated currents, demonstrating that it is a bona fide mechanically activated ion channel^89^. In the family of TMEM63 proteins, TMEM63A, TMEM63B, and TMTM63C are mammalian orthologs of *A. thaliana* OSCAs (AtOSCAs)^90^. Thus, TMEM63 proteins from different species can potentially represent mechanosensitive ion channels with mechanisms and functions similar to those of AtOSCAs.

#### Structure

High-resolution cryo-EM structures of AtOSCA1.1, AtOSCA3.1, and AtOSCA1.2 have been determined and revealed to be dimeric, with each subunit consisting of 11TM helices^23,91–93^ (Fig. 1n–p). Both subunits are separated by a large central cavity filled with lipids, which may aid in the dimeric complex’s stability or gating^23,91,92^. The cytosolic domain of OSCA is mainly composed of the intracellular TM2-TM3 loop and the C-terminus which possesses a unique structural feature with an elongated two-blade propeller shape that is parallel to the plasma membrane^23,91,92^. Interestingly, despite their low sequence similarity, OSCAs have structural similarity with the calcium-activated chloride channel TMEM16 family, which forms a homodimer^23,91–93^. As TMEM16A, which has two pores within each functional protein, one from each subunit, OSCA also has two ion conduction pathways lined by TM3-TM7 in each subunit^23,91,92^. Below the hydrophobic neck, Glu462 at the base of TM5 in OSCA1.1 and Glu531 of TM6 in OSCA1.2 protrude into the hydrophilic pore cavity and exert a significant impact on single-channel conductance, suggesting that this region indeed contributes directly to the ionic pore^23,91^. Although these results reveal the overall structure and ion conduction pathway, the detailed atomic-level molecular mechanisms of OSCAs as mechanosensitive channels are still lacking and should be elucidated in future investigations.

#### Physiological and pathophysiological roles

Genetic deletion of OSCA1 in *A. thaliana* results in impaired osmotic Ca^2+^ signaling in guard cells and root cells and restricted regulation of transpiration and root growth in response to osmotic stress^13^. Overexpression of TMEM63B enhances Ca^2+^ influx across the plasma membrane and cell migration in HEK293T cells^94^. TMEM63B is highly expressed in inner ear hair cells and plays a role in osmosensation as a swelling-activated nonselective cation channel^95^. TMEM63B is required for the survival of outer hair cells and hearing via mediation of Ca^2+^-dependent regulatory volume decrease under hypotonic stress^95^. TMEM63A is abundantly expressed in nonpeptidergic nociceptors and modulates chronic post‑amputation pain in synchrony with local macrophages^96^. TMEM63A is enriched in oligodendrocytes, and heterozygous missense mutations in this gene, such as Gly168Glu, Ile462Asn, and Gly567Ser, lead to infantile disorders that resemble transient hypomyelination^97^. Heterozygous variants of TMEM63B are associated with severe early-onset developmental and epileptic encephalopathy and progressive neurodegenerative brain changes^98^. TMEM63C plays a major role in mediating the glomerular filtration barrier in zebrafish, and its expression is deficient in patients with focal segmental glomerulosclerosis^99^. Taken together, the abundant associations of the OSCA family of stretch-activated ion channels with numerous diseases highlight its importance throughout the body.

### TMEM150C (Tentonin3)

#### Electrophysiological properties

TMEM150C has been identified as a mechanosensitive ion channel that is responsible for slowly adapting-type mechanically activating (SA-type MA) currents in DRG neurons^14^. It was renamed Tentonin3, from the Greek ‘tentono’, meaning to stretch^14^. Heterologous expression of TMEM150C results in robust currents induced by mechanical step stimuli, with slow inactivation, which is distinct from the rapidly inactivating PIEZO1-mediated current^14^. Its current is inhibited by gadolinium, FM1-43, and GsMTx4, which are known blockers of mechanosensitive channels^14^. Genetic ablation of TMEM150C significantly reduces SA-type MA currents in DRG neurons^14^. TMEM150C in the lipid bilayer elicits spontaneous and stretch-activated channel currents^100^. These results suggest that TMEM150C has a pore-forming subunit that is sensitive to membrane stretching. Furthermore, it has been postulated that the N- or C-terminus of TMEM150C might be involved in the gating process based on the observation that deletion of the N- or C-terminus independently causes a marked reduction in MA currents^100^. However, it is still unclear how mechanical transduction causes channel activation.

#### Structure

A high-resolution structure is not yet available for TMEM150C. Recently, *Pak* et al. suggested a tetrameric composition of TMEM150C proteins based on western blot analysis of cross-linked TMEM150C^100^. Furthermore, once the optimal tetrameric TMEM150C conformation was determined via a simulation program, four types of tetrameric structures were generated^100^. However, the precise and detailed molecular mechanisms for mechanical activation require cryo-EM or crystal structure analysis.

#### Physiological and pathophysiological roles

TMEM150C is expressed not only in DRG neurons but also in muscle spindles, in which genetic deletion of TMEM150C leads to a reduction of motor coordination through muscle spindle afferents^14^. TMEM150C, expressed in baroreceptors, plays an essential role in detecting blood pressure as a mechanosensitive channel in the aortic arch. Genetic ablation of TMEM150C in mice causes ambient hypertension, tachycardia, AP fluctuations, and impaired baroreflex sensitivity^101^. Additionally, TMEM150C is expressed in pancreatic β cells and contributes to glucose-stimulated insulin release^102^. As a mechanosensitive ion channel that is expressed in several critical organs of the body, TMEM150C may be associated with various human diseases, necessitating further investigation of the physiological and pathological roles of this protein.

### TMEM43 (Gapjinc)

#### Electrophysiological properties

TMEM43 has been identified as a nonselective cation channel that is permeable to Na^+^, K^+^, and Cs^+^ ions^103^. Its topology is predicted to include 4 TMs and 1 intramembrane domain with extracellular N- and C-termini^104^. Heterologously expressed TMEM43 mediates passive conductance-like currents, which are potently blocked by the nonselective cation channel blocker GdCl_3_^103^. Furthermore, TMEM43-mediated currents are inhibited in a dose-dependent manner by the extracellular pH, such that reducing the pH from 8 to 5.5 gradually decreases the current passing through the channel^103^. In glia-like support cells of the cochlea, the TMEM43 protein interacts with the gap junction connexin subunits Connexin26 and Connexin30 and functions as an essential component of the passive conductance current; gene silencing of TMEM43 significantly eliminates carbenoxolone-, Gd^3+^- and low pH-sensitive passive conductance currents^104^. An additional report demonstrated that TMEM43 is essential for mediating the passive conductance current in glia-like support cells by interacting with the TASK-1 protein, which is a two-pore-domain potassium channel^105^. Interestingly, although TMEM43 is highly expressed in hippocampal astrocytes and strongly interacts with Connexin43, it does not contribute to the passive conductance current in those cells^103^. This finding is consistent with previous observations that gap junction channels do not contribute to the passive conductance current in hippocampal astrocytes. Finally, the purified TMEM43 protein in a lipid bilayer displays stochastic single-channel openings^103^. These lines of evidence strongly suggest that TMEM43 is a bona fide cation channel that strongly interacts with gap junction channels; thus, the name Gapjinc (Gap junction interacting channel) is appropriate^103^.

#### Structure

A high-resolution structure is not yet available for TMEM43. Although the monomeric architecture of TMEM43 is available through AlphaFold2^106^, its monomeric structure contains an incomprehensible scrambled intramembrane domain. Therefore, cryo-EM or crystal structure analysis is needed to determine the detailed structural correlates of the ion channel functions.

#### Physiological and pathophysiological roles

TMEM43 has been implicated in various human diseases, such as cancer^107,108^, heart disease^109–111^, and hearing loss^104^. Upregulation of TMEM43 in glioblastoma accelerates tumor survival and progression through the interaction of TMEM43 with CARD-containing MAGUK protein 3 (CARMA3) during EGFR-induced NF-κB activation^107^. In addition, TMEM43 plays a critical role in promoting pancreatic cancer progression by stabilizing PRPF3 and regulating RAP2B/ERK^108^. Mutation of TMEM43 (p.Ser358Leu) causes arrhythmogenic right ventricular cardiomyopathy^110,111^, which is a heritable cardiomyopathy that is being increasingly recognized as a cause of sudden cardiac death by increasing the stiffness of the cell nucleus and potentially causing massive loss of cardiomyocytes^111^.

Genetic studies in humans have found that the point mutation of TMEM43 causes autosomal dominant auditory neuropathy spectrum disorder, which is a hearing impairment disorder associated with an inability to discriminate speech, despite relatively preserved pure-tone detection thresholds^104,112^. A nonsense mutation in TMEM43 (p.Arg372Termination) has been identified as a novel deafness gene in Chinese and Korean families^104^. In the cochlea, this mutation disrupts channel activity^103^ and connexin-linked functions in glia-like support cells and consequently disrupts the homeostasis of the inner ear for hearing^104^.

Recently, it has been demonstrated that TMEM43 plays an important role in gap junction networks in the brain. Astrocytic dye diffusion through the gap junction-coupled syncytium decreases with decreasing K^+^ uptake, while neuronal excitability increases in the hippocampus of TMEM43 KO mice^15,103^. Due to these impairments, TMEM43 KO mice exhibit significantly impaired late long-term potentiation and hippocampus-dependent memory^15, 103^. Taken together, these findings indicate that TMEM43 is an important protein that is broadly expressed and interacts with gap junction proteins, plays important gap junction-related functions, and is associated with various diseases, such as arrhythmogenic right ventricular cardiomyopathy, auditory neuropathy spectrum disorder, and memory impairments.

## Conclusion

Recent studies have established that many TMEM proteins are ion channels and play key roles in various physiological and pathophysiological processes. Dysfunction abnormalities can cause diseases, including Parkinson’s disease, cancer, osteogenesis imperfecta, and hypomyelination in infants as well as cardiomyopathy and auditory neuropathy spectrum disorders. Therefore, discovering the physiological and pathophysiological functions of novel TMEM ion channels will spur new developments in therapeutic drugs or tools for various pathologies that have not yet been identified as channelopathies. A better understanding of the atomic resolution structures of these ion channels is an absolute prerequisite for effective drug development. Here, we summarize the unique functional properties and structures of recently discovered TMEM proteins in anticipation of new discoveries for the remaining TMEM proteins of unknown function.
